# Effects of Perfluorocarbons on surfactant exocytosis and membrane properties in isolated alveolar type II cells

**DOI:** 10.1186/1465-9921-11-52

**Published:** 2010-05-09

**Authors:** Andreas Wemhöner, Irmgard Hackspiel, Nina Hobi, Andrea Ravasio, Thomas Haller, Mario Rüdiger

**Affiliations:** 1 University Hospital Dresden, Department for Pediatric Intensive Care and Neonatology, Technical University Dresden, Germany; 2 Department of Pediatrics, Neonatology; Innsbruck Medical University, Austria; 3 Department of Physiology and Medical Physics, Innsbruck Medical University, Austria

## Abstract

**Background:**

Perfluorocarbons (PFC) are used to improve gas exchange in diseased lungs. PFC have been shown to affect various cell types. Thus, effects on alveolar type II (ATII) cells and surfactant metabolism can be expected, data, however, are controversial.

**Objective:**

The study was performed to test two hypotheses: (I) the effects of PFC on surfactant exocytosis depend on their respective vapor pressures; (II) different pathways of surfactant exocytosis are affected differently by PFC.

**Methods:**

Isolated ATII cells were exposed to two PFC with different vapor pressures and spontaneous surfactant exocytosis was measured. Furthermore, surfactant exocytosis was stimulated by either ATP, PMA or Ionomycin. The effects of PFC on cell morphology, cellular viability, endocytosis, membrane permeability and fluidity were determined.

**Results:**

The spontaneous exocytosis was reduced by PFC, however, the ATP and PMA stimulated exocytosis was slightly increased by PFC with high vapor pressure. In contrast, Ionomycin-induced exocytosis was decreased by PFC with low vapor pressure. Cellular uptake of FM 1-43 - a marker of membrane integrity - was increased. However, membrane fluidity, endocytosis and viability were not affected by PFC incubation.

**Conclusions:**

We conclude that PFC effects can be explained by modest, unspecific interactions with the plasma membrane rather than by specific interactions with intracellular targets.

## Introduction

Perfluorocarbons (PFC) that are used to improve gas exchange in diseased lungs with disturbed pulmonary surfactant system affect various cell types [1-4]. Thus, an effect on alveolar type II (ATII) cells and surfactant metabolism can be expected, data, however, are scarce and inconsistent. Some authors did not find an effect of PFC on pulmonary surfactant content [5-7]. Other studies suggested that PFC affect surfactant production, however, the effect depended on the type of PFC used [8,9]. Steinhorn and co-workers showed an increased amount of pulmonary choline in PFC treated rabbits, suggesting an enhanced surfactant production during liquid ventilation [10]. According to data of our group, PFC might increase surfactant exocytosis in isolated ATII cells [11], however, not in liquid ventilated rats [12].

Whereas most studies investigated the effect on spontaneous surfactant exocytosis, data are missing that quantify the effect of PFC on stimulated exocytosis in isolated type II cells. Surfactant exocytosis can be stimulated by different substances. Adenosintriphosphat (ATP) is a potent stimulant of surfactant exocytosis in vitro. ATP activates protein kinase C (PKC) and increases intracellular Ca^2+^-concentration. Phorbol 12-myristate 13-acetate (PMA) passes cellular membranes and activates intracellular PKC. Ionomycin binds Ca^2+ ^in the extracellular space, diffuses into the cell where the bound Ca^2+ ^is released, leading to an increase of intracellular Ca^2+ ^concentration [13,14].

Interaction of PFC with cellular processes seem to depend on vapor pressure, an effect that is most likely mediated by a stabilization of cellular membranes [15]. Surfactant exocytosis requires a fusion of lamellar bodies with the cellular membrane [13,16]. Thus, we hypothesized that PFC with different vapor pressures affect surfactant exocytosis. To test the hypothesis, a recently developed assay for exocytosis in ATII cells was used [17]. Isolated cells were exposed to two PFC with different vapor pressures and surfactant exocytosis was stimulated by ATP, PMA or Ionomycin. To further understand the effect of PFC, the impact of PFC on cellular membrane properties was studied.

## Materials and methods

### ATII cells and reagents

Experiments were performed using freshly isolated alveolar type II (ATII) cells. Animal care and use were approved by the Institutional Animal Care and Use Committee of the Innsbruck Medical University (ZI A 07/3456). The cells were isolated from male Sprague-Dawley rats (180-200 g) according to methods of Dobbs et al. [1,18] with minor modifications [13]. Isolated cells were seeded in 200 μl of DMEM at high density (10^6 ^cells/ml) in 96-well tissue culture plates with flat bottoms (Sarstedt, Austria) for multiplate reader measurements. Cells were incubated in DMEM with 5 mM glucose, supplemented with 24 mM NaHCO_3 _and 10% FCS in a humidified atmosphere with 5% CO_2 _at 37°C. After 48 hours, medium was removed from the cells by washing twice with serum-free DMEM.

For fluorescence anisotropy measurements A549 cells were cultured in F-12 Ham's medium (Invitrogen, Austria) supplemented with 10% heat-inactivated FBS, 100 U/ml of penicillin, and 100 μg/ml streptomycin at 37°C in a humidified incubator with 5% CO_2_. A549 cells were obtained from ATCC (American Tissue Culture Collection, USA).

Ringer solution contained, in mM: 140 NaCl, 5 KCl, 1 MgCl_2_, 2 CaCl_2_, 5 glucose, and 10 HEPES (pH 7.4 at 25°C). LysoTracker Green DND-26 (LTG) was purchased from Molecular Probes (Austria), Brilliant Black (BB) from MP Biomedicals (Germany). All other chemicals were obtained from Sigma-Aldrich (Austria). All experiments were performed at room temperature.

### Perfluorocarbons

Two different PFC were obtained from F2 chemicals (Lancashire, UK). They differ with respect to vapor pressure (Table 1). Whereas Perfluorodecalin (PFD) has a low vapor pressure, PP2 has a high one.

**Table 1:** Physicochemical properties of PFC were obtained from F2 chemicals

	Perfluorodecalin (PFD)	Flutec™ PP2; Perfluoromethylcyclohexan (PP2)
Formula	C_10 _F_18_	C_7 _F_14_

Vapor pressure, mbar	6.25	141

Viscosity, mm^2^/s	2.61	0.88

Density, kg/l	1.92	1.79

Boiling point (°C)	142	76

Since PFC are water insoluble and thus cannot been used for cellular incubation, PFC-in-DMEM suspension were used for subsequent experiments [19]: DMEM was mixed with PFC at a ratio (v/v) of 9:1. The mixture was exposed to ultrasonic energy for 10 min in a transonic analogous ultrasonic unit (35/kHz, 70 HF peak/W ELMA Singen, Germany). After mixing, two distinct liquid phases can be separated. The supernatant representing the PFC-in-DMEM suspension and the lower liquid phase is still consist PFC.

To test stability of suspensions of PFC in DMEM, they were pipetted onto glass cover slips. Images were taken with a 100× Plan Apochromat DIC, NA 1.4, (Zeiss) and analyzed using ImageJ. Number of droplets and size distribution were measured over a period of 6 hours. No differences in droplet size or number were found suggesting a relatively high stability of PFC-in-DMEM suspension (data not shown).

### Surfactant exocytosis assay

To study the effects of PFC on surfactant exocytosis, a fluorescence based microplate assay for exocytosis in adherent ATII cells was used as recently described in detail [17]. In short, isolated and adherent cells were incubated with DMEM and 10% PFC for 2, 4 and 6 hours, respectively. After incubation with PFC wells were rinsed twice with Ringer solution and cells were incubated with 500 nM LTG at 37°C in DMEM without FCS. After 30 minutes, the wells were rinsed twice with Ringer solution to remove floating cells and remaining dye. Thereafter, LTG is exclusively located within the Lamellar Bodies (LBs). The wells were finally filled with 200 μl of the Ringer solution containing BB at a concentration of 2 mg/ml.

To estimate exocytosis, the level of intracellular LTG fluorescence was measured by a microplate reader (GENios Plus; Tecan, Austria). Briefly, the microplate reader collects the emitted light from LTG-loaded cells. This signal correlates with the amount of intracellular LBs, and declines rapidly when the cells activate these vesicles to release their contents, including the water soluble LTG, into the extracellular space. An increased rate of vesicle fusion results in a time-dependent deviation of the measured signals between treated and untreated cells. Superimposed to all measurements is an almost linear fall in background fluorescence which is due to a combination of constitutive (= spontaneous) exocytosis, photobleaching, quenching effects and others.

**Figure 1:**
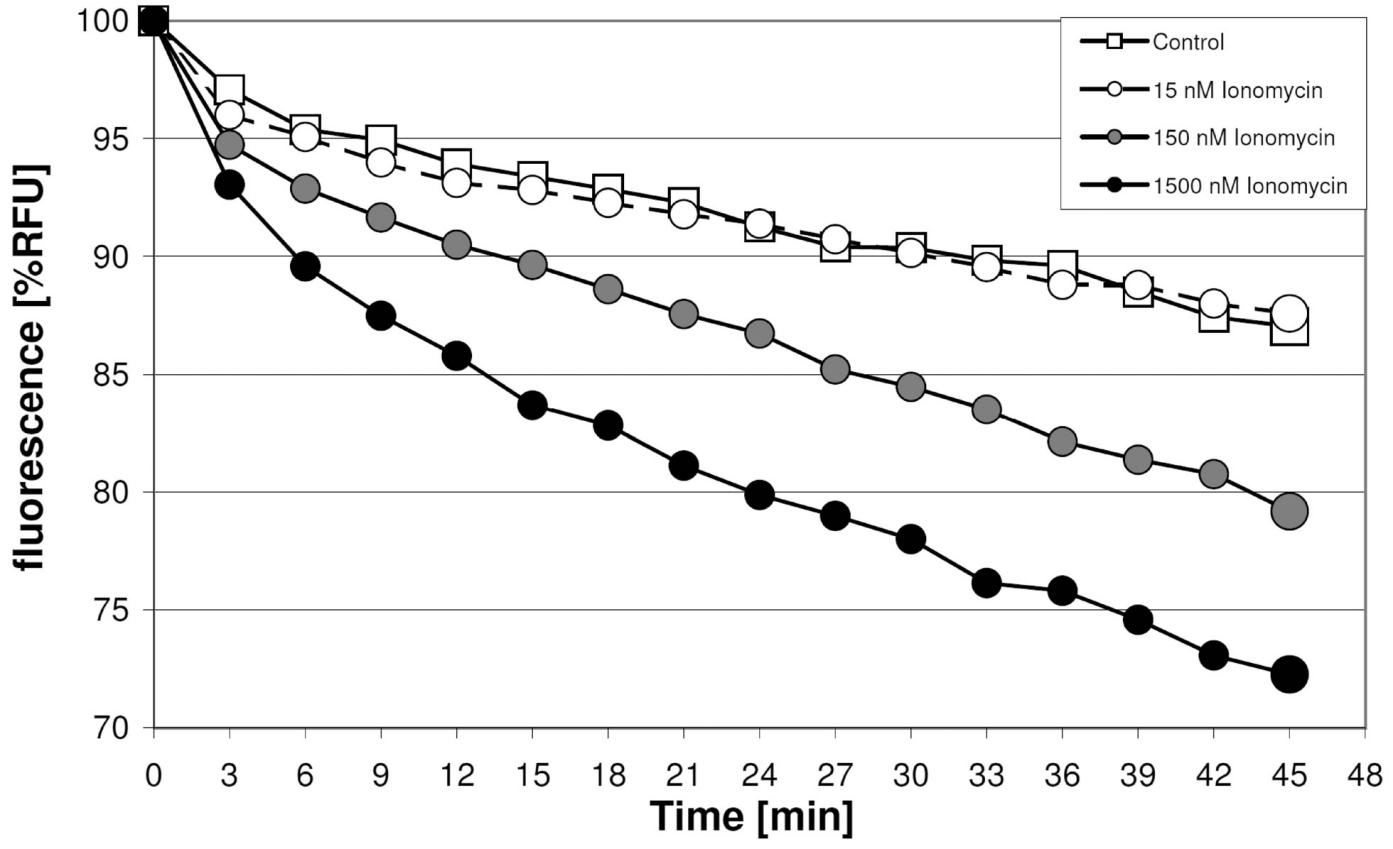
**Ionomycin effect on the rate of vesicle fusion in ATII cells**. Relative fluorescence units (RFU) between single wells, at time 0, were converted to 100%. Carrier alone (controls) and Ionomycin in different concentrations were applied thereafter, between 1^st ^and 2^nd ^data point. Enlarged symbols at the end of the recordings indicate the endpoints taken for all subsequent analyses.

Cells were excited by 3 consecutive light flashes directed through a 485-nm band pass filter onto the bottom of the culture plates. Three flashes were used as this mode of measurement provided the best results regarding signal stability and sample throughput. Emission light was collected by a 535-nm band pass filter from the bottom of the plates and integrated over 2 ms for each well. Fluorescence values were obtained every 3 min for a period of 45 min. The reduction in fluorescence denotes the rate of exocytosis (Figure 1).

To study the effect of PFC on stimulated surfactant exocytosis cells were treated with either ATP, PMA or Ionomycin. In short, 150 μM of ATP, 1.5 μM of PMA or 1.5, 15, 150 and 1500 nM of Ionomycin were added to Ringer solution containing BB per well.

The Ionomycin-induced surfactant exocytosis has not been tested with the present assay previously. As shown in Figure 1, Ionomycin had a dose dependent effect. In cells that were stimulated with 15 nM Ionomycin surfactant exocytosis did not differ from non-induced exocytosis. After addition of 150 nM and 1500 nM Ionomycin surfactant exocytosis was 8% (p = 0.001) and 15% (p = 0.0001) higher than the spontaneous rate, respectively.

### Test for PFC effects on plasma membrane properties

PFC-induced alterations in general properties of the cell membrane were studied in an assay using FM 1-43. This styryl dye specifically inserts into the outer leaflet of cell membranes where its fluorescence emission is considerably enhanced. Therefore, FM 1-43 is one of the preferred dyes for stable cell membrane labeling [20]. However, despite its cationic head groups at neutral pH, a very slow permeation of FM 1-43 into cells occurs [20,21], which may be associated with alterations in the physical and biochemical membrane properties or drug-induced membrane perturbations.

**Figure 2:**
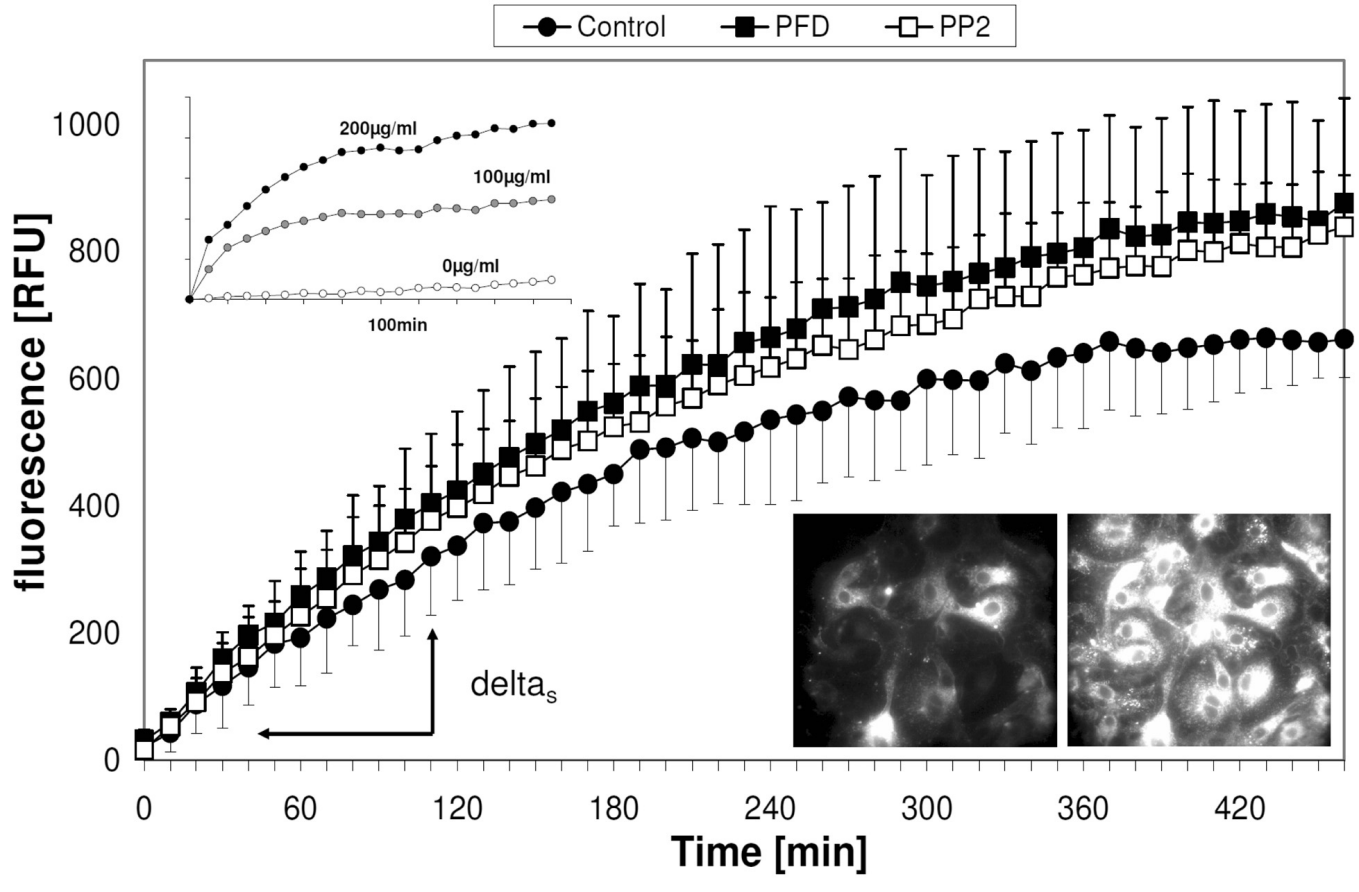
**Kinetics of FM 1-43 uptake into cells**. Delta was expressed as relative fluorescence units (RFUs) and represents the rate of FM 1-43 uptake used for the values shown in Table 2. Images: Fluorescence microscopy demonstrates intracellular FM 1-43 uptake by ATII cells after 30 min and 3 hours incubation. Note that PFC droplets are not present. Inset: Rate of increase intracellular fluorescence intensity of FM 1-43 after concentration-dependent ATII cell incubation with β-Escin. These experiments demonstrate the sensitivity of this assay with respect to membrane perturbations. Data of FM 1-43 cell uptake were expressed as relative fluorescence units (RFUs).

The measurements were performed by adding 10 μM of FM 1-43 and 1 mg/ml BB to each well. Since FM 1-43 has a low fluorescence in water, and its binding to lipid-water interfaces is reversible, stable and specific signals from the cell membranes necessitate the continuous presence of this dye in the cell supernatants, and of BB (or any other quencher) to block fluorescence of free dye molecules in suspension. Immediately after addition of FM 1-43 and BB, the plate was inserted into the plate reader and a kinetic cycle started. Measuring conditions were: _exc _= 485 nm, _em _= 595 nm, bottom reading mode, T = 37°C, measuring interval every 10 min for in total 8 hrs. The recording conditions were chosen to follow the initial rates as well as the steady-states of fluorescence changes. Feasibility and sensitivity of the assay was validated by using the pore-forming drug β-Escin [22]; Inset in Figure 2: The strong, dose- and time-dependent rise in fluorescence is due to the abundance of intracellular lipid/water-interfaces, into which FM 1-43 can intercalate, and an increase in drug-induced membrane perturbations.

**Table 2:** PFC effects on cells and cellular membranes

	PFD	PP2
Rate of FM 1-43 uptake	124 ± 17%*	121 ± 7%*

Total FM 1-43 uptake	128 ± 10%*	123 ± 14%*

Total LY uptake	94 ± 15%	105 ± 16%

Trypan Blue	101 ± 6%	106 ± 6%

DPH fluorescence anisotropy	0.105 ± 0.03	0.102 ± 0.03

Effects of PFC after 6 hours incubation on permeability properties (rate and total uptake of FM 1-43), endocytosis (total LY uptake), vitality (Trypan Blue) and membrane fluidity (DPH fluorescence anisotropy). Shown are relative data, mean and standard deviation, of PFC incubated ATII cells vs. cells without PFC incubation (= 100%; n = 3 independent rat preparations with 5 samples each; *p < 0.05). Anisotropy measurements were performed with A549 cells and presented are absolute data (*r*_ss _untreated control cells = 0.119 ± 0.03; [24].

Further tests for changes in membrane properties were performed measuring fluorescence anisotropy [23] with the membrane probe 1,6-diphenyl-1,3,5-hexatriene (DPH); [24] on a Spex spectrofluorimeter (SPEX Fluorolog 2 spectrofluorimeter (CM-1, Edison, NJ, USA) equipped for polarization measurements). Fluorescence polarization depends on the molecular mobility of a fluorophore in solution. The higher it is, the lower will be the polarization (anisotropy) of the emitted light. Thus, this technique has been widely used to measure the fluidity of biological membranes.

After 6 hours PFC incubation suspensions of A549 cells, harvested by trypsinization from petri-dishes, were loaded for 5 min with 10 μM DPH, followed by a washing step before measurement in quartz cuvettes. ATII cells turned out to accumulate DPH primarily in LBs, outranging the signal from the plasma membrane manifold. Therefore, we used A549 cells which do not contain LBs despite of being of type II cell origin. Measurement conditions on the spectrofluorimeter were: _exc _= 360 nm, _em _= 430 nm, T = ambient, 5 measurements/sample, n = 6. Data were calculated according to the formula given by Sousa et al. [24].

### Measurements of endocytosis

For measurements of endocytosis we used the fluid phase marker Lucifer Yellow (LY). LY was applied at 4 mg/ml in FCS-free DMEM at 37°C for the indicated periods of time. Prior to measurements, the wells were washed 3 times in Ringer solution, and BB (2 mg/ml) was added to quench any remaining extracellular LY fluorescence. The plate was inserted into the plate reader and end-point measurements were taken at _exc _= 450 nm and _em _= 535 nm in the bottom reading mode. Specificity of LY staining of cells was verified by fluorescence microscopy (not shown).

### Cell viability

The amount of viable cells was measured by the trypan blue exclusion method. Cells were harvested with trypsin-EDTA. After neutralization of trypsin-EDTA with DMEM containing 10% FCS, cell number and viability were examined by light microscopy.

### Statistics

**Figure 3:**
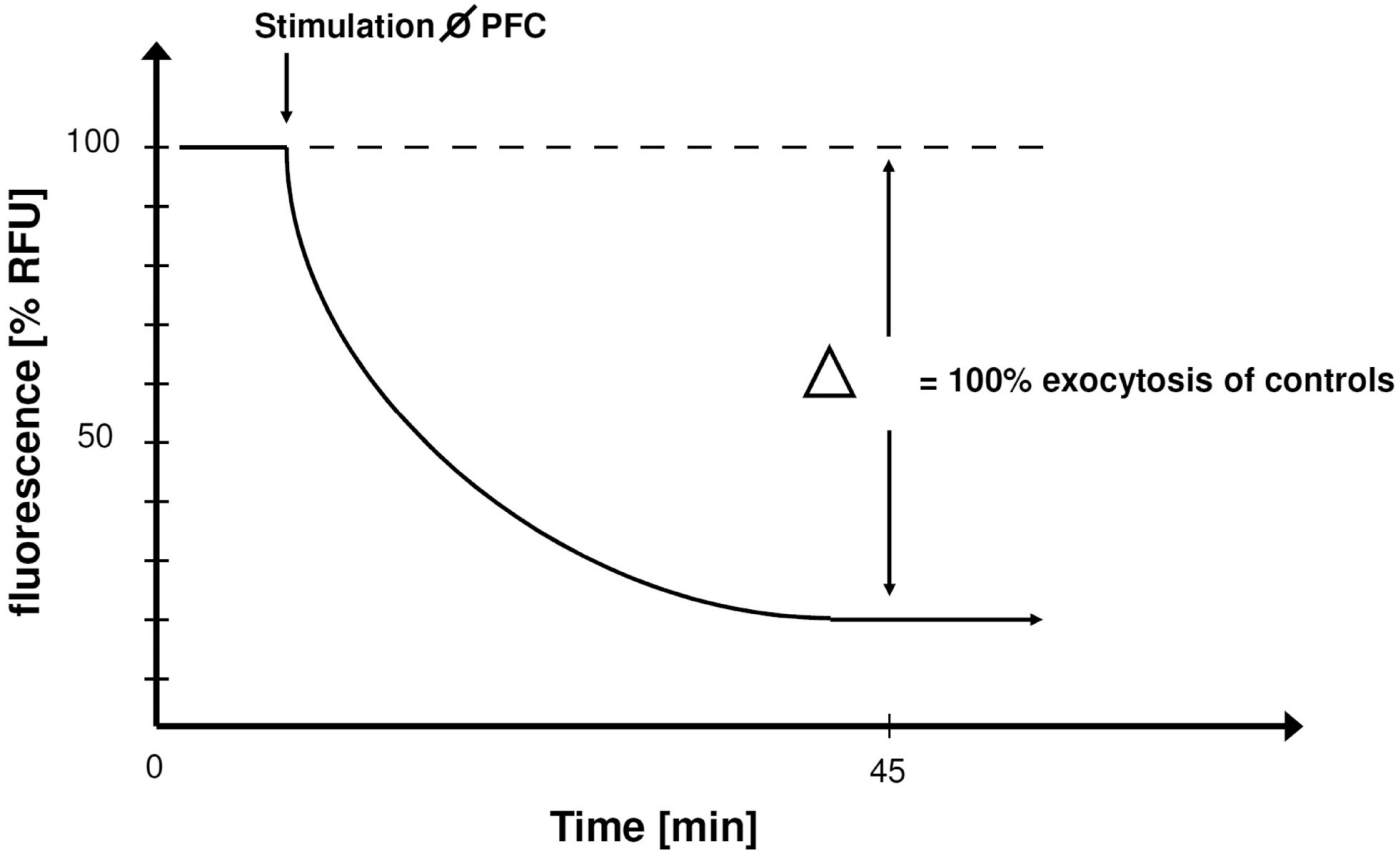
**Explanation of data analysis for exocytosis measurements**. Exocytosis of control cells (PFC-free) was calculated as the % decline of RFU 45 min after the respective stimulation (arrows). Values from single experiments were then normalized by their average decline. Thus, control values in Figures 4, 5, 6 and 7 are shown as 100% ± SD. Data (RFU-decline) from PFD treated cells were normalized to the average decline of those controls. Data were compared by t-test in which the statistical significance was taken as p < 0.05.

**Figure 4:**
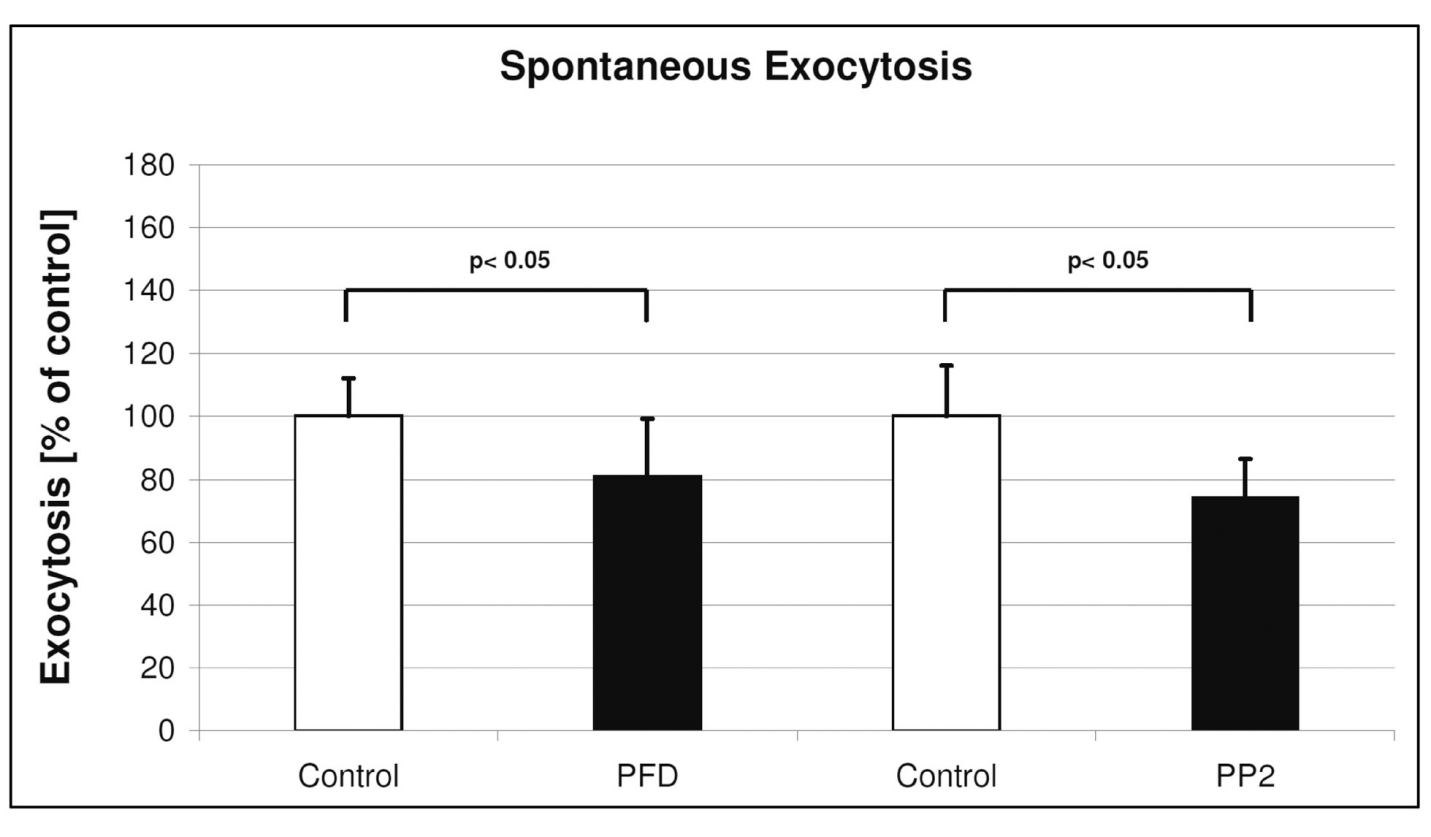
**Effects of PFC on spontaneous exocytosis**. Shown are changes on time point 45 (min) in LTG fluorescence after 6 hours PFC incubation (mean and SD of n = 3 independent rat preparations with 3 samples each). Data were compared by *t*-test in which the statistical significance was taken as *p *< 0.05.

**Figure 5:**
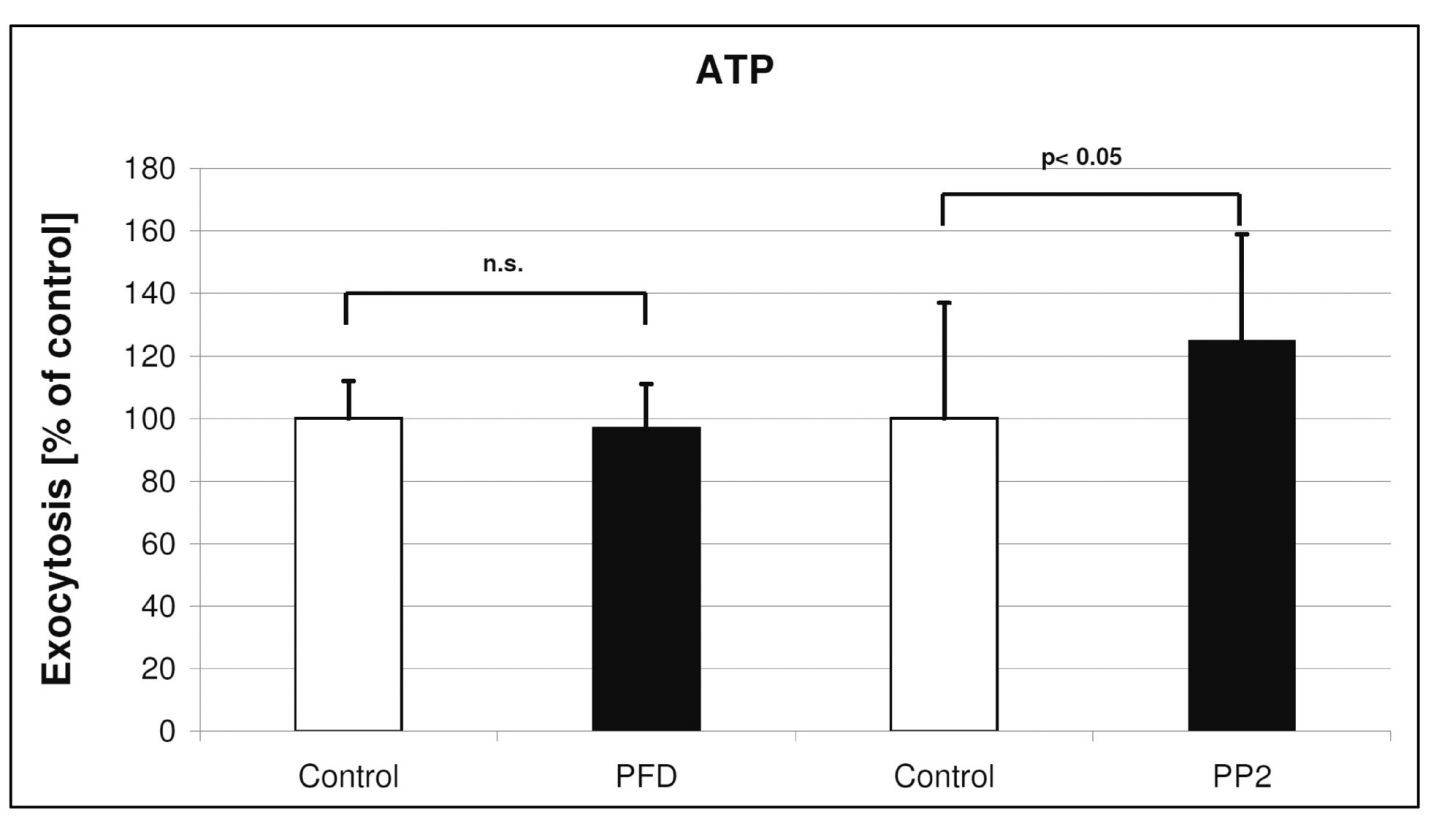
**Effects of PFC on ATP stimulated (150 μM) exocytosis**. Shown are ATP-evoked changes on time point 45 (min) in LTG fluorescence after 6 hours PFC incubation (mean and SD of n = 3 independent rat preparations with 3 samples each). Data were compared by *t*-test in which the statistical significance was taken as *p *< 0.05.

**Figure 6:**
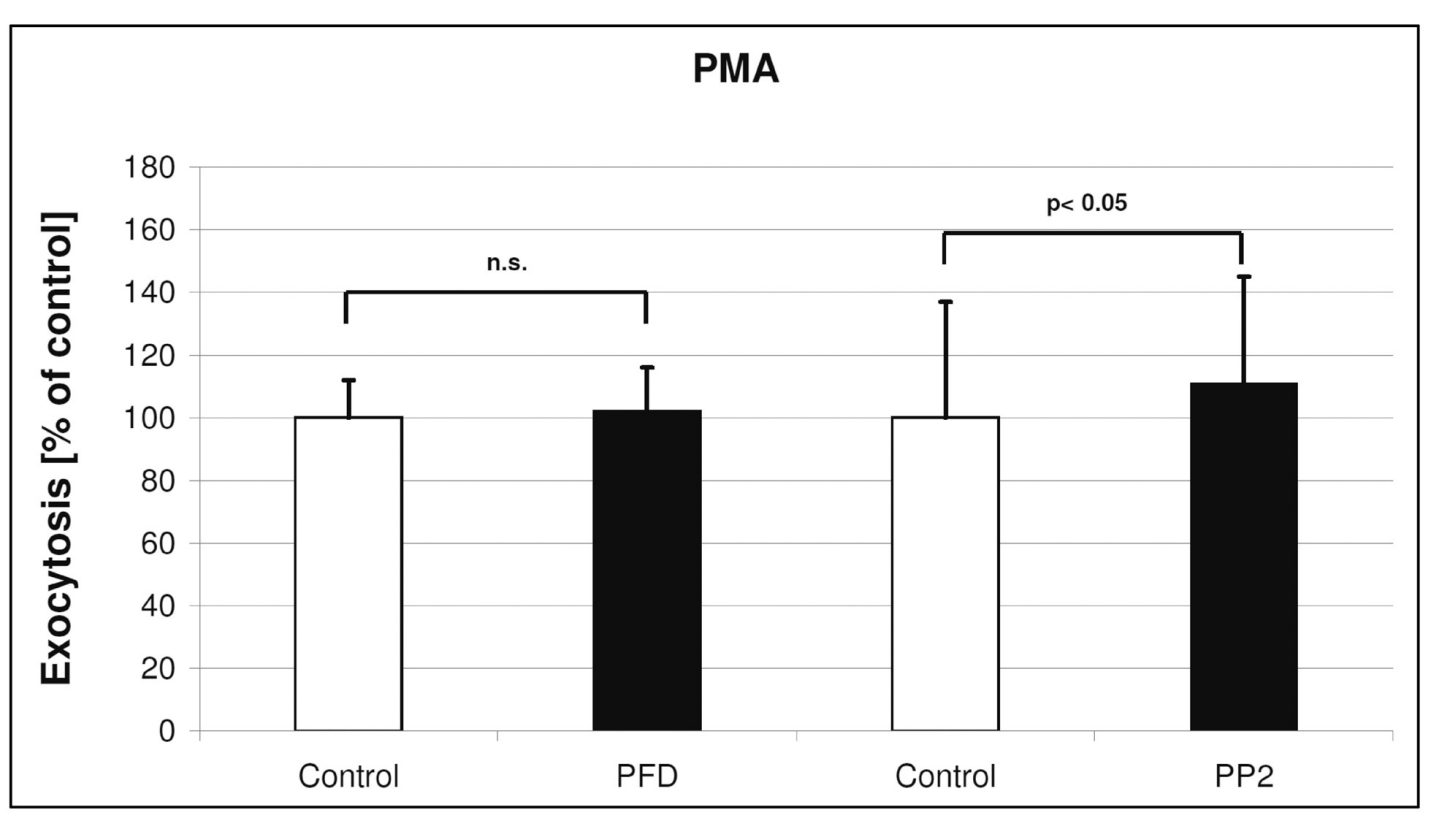
**Effects of PFC on PMA stimulated (1.5 μM) exocytosis**. Shown are PMA-evoked changes on time point 45 (min) in LTG fluorescence after 6 hours PFC incubation (mean and SD of n = 3 independent rat preparations with 3 samples each). Data were compared by *t*-test in which the statistical significance was taken as *p *< 0.05.

**Figure 7:**
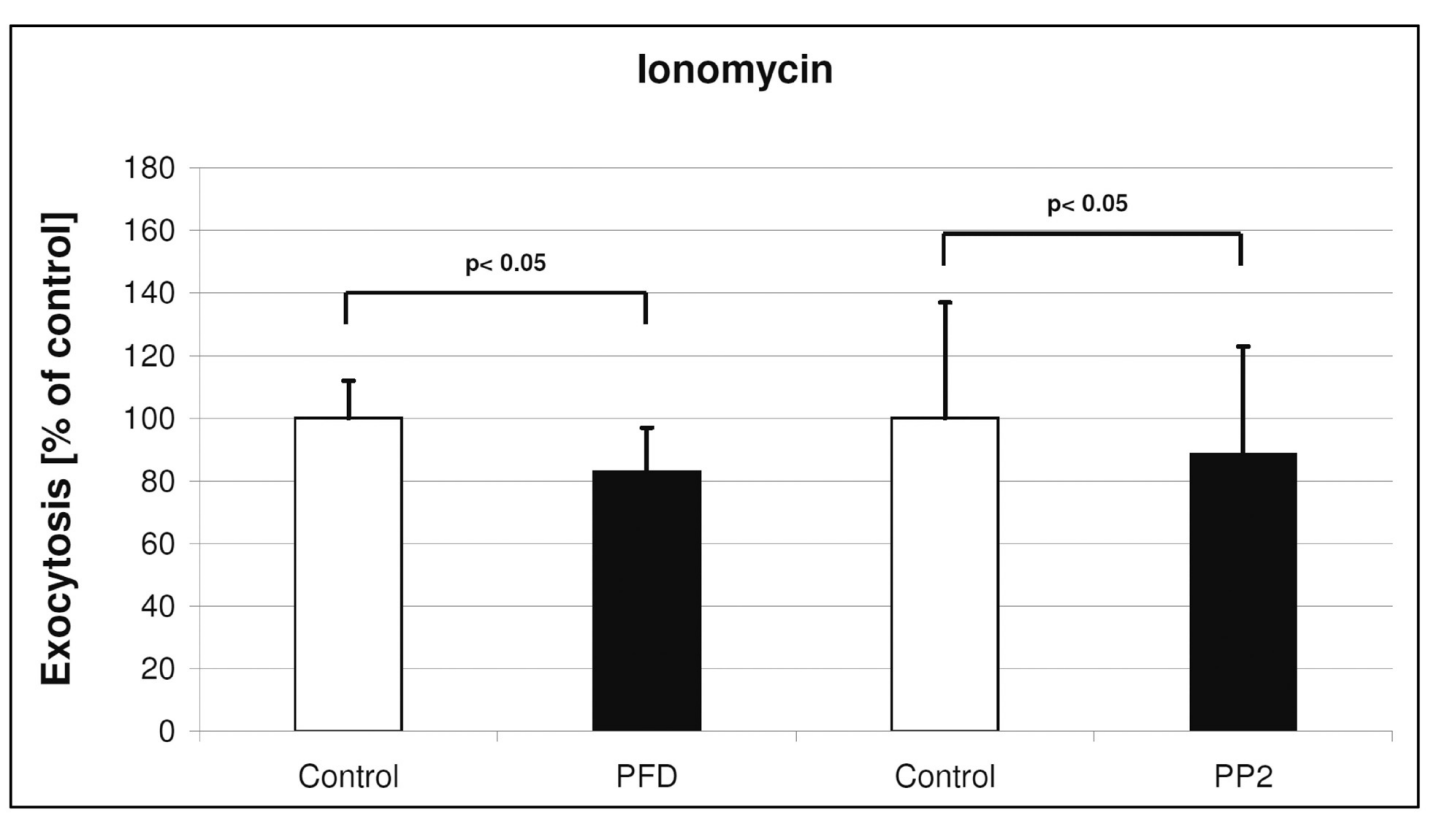
**Effects of PFC on Ionomycin stimulated (1.5 μM) exocytosis**. Shown are Ionomycin-evoked changes on time point 45 (min) in LTG fluorescence after 6 hours PFC incubation (mean and SD of n = 3 independent rat preparations with 3 samples each). Data were compared by *t*-test in which the statistical significance was taken as *p *< 0.05.

Fluorescence intensities from individual wells were background corrected by subtracting the signals obtained from unlabeled but otherwise identically treated wells. To avoid miss-calculations due to different loadings of cells and to compensate for differences in cell densities, data were expressed as normalized values (100% RFU). The mean change in signal intensity of all control cells during 45 min was calculated and taken as 100% exocytosis (Figure 3). Thereafter, the difference from this mean change in signal intensity was calculated for each individual well (included the treated but also the control cells). Thus, control values show a statistical distribution around 100% e.g. Figures 4, 5, 6 and 7.

For each experimental set, data from at least 3 independent animal preparations were used to calculate the mean ± SD. Data were compared by *t*-test, a p value of < 0.05 was considered as statistically significant.

## Results

### Effect of PFC on spontaneous surfactant exocytosis

The intracellular amount of LTG was significantly higher after 6 hours of PFC incubation when compared with untreated control cells. When compared with control cells, the surfactant exocytosis was decreased in PFD and PP2 treated cells. Thus, PFC incubation was associated with a significant reduction in spontaneous surfactant exocytosis (Figure 4).

### Effect of PFC on stimulated surfactant exocytosis

To investigate whether PFC affect stimulated surfactant exocytosis, we used ATP, PMA, or Ionomycin to induce surfactant exocytosis.

After ATP stimulation, surfactant exocytosis was increased by ~7%, similar to a previous report [17]. PFD incubation did not affect the ATP-stimulated rate of exocytosis, whereas PP2 incubation was associated with a significant increase in ATP-stimulated exocytosis (Figure 5). PMA incubation increased surfactant exocytosis by ~11% over controls [17]. PFD incubation did not affect the PMA-stimulated exocytosis, whereas PP2 incubation was associated with a small but significant increase in PMA-stimulated exocytosis (Figure 6).

Stimulation of ATII cells with 1500 nM Ionomycin increases surfactant exocytosis by 14% (Fig. 1). PFD and PP2 incubation did prevent the Ionomycin-stimulated exocytosis (Figure 7).

### Morphological analysis

PFC are known to interact with cellular membranes. Thus, it was tested, whether membrane properties or integrity are affected by PFC, using various approaches.

**Figure 8:**
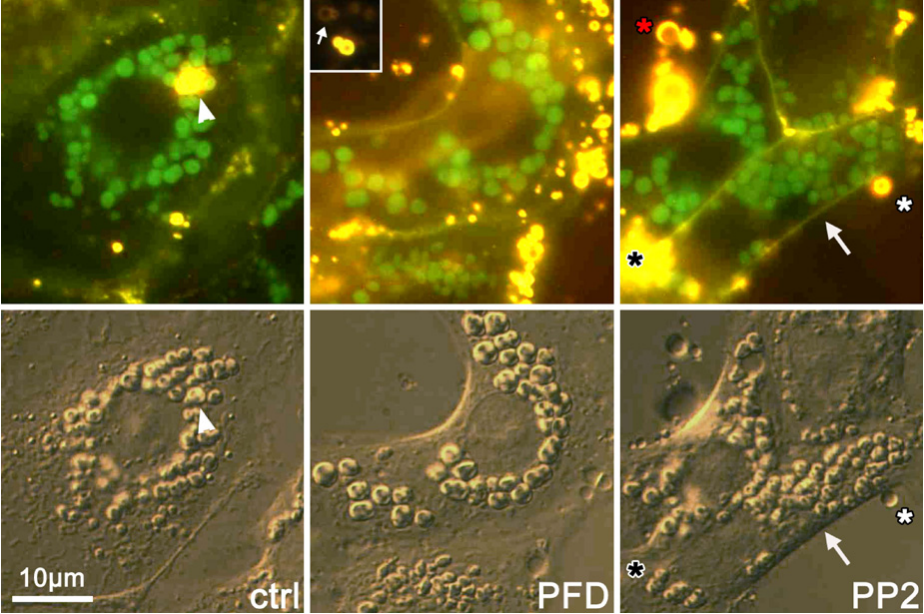
**ATII cells imaged in parallel by fluorescence (upper panel) and DIC (lower panel) in the presence or absence (ctrl) of PFC**. Green fluorescence (LTG) denotes intracellular LBs, and shows a clear correlation with DIC. FM 1-43 (yellow, 1 μM) heavily stains exocytosed (arrowhead) or extruded surfactant (black asterisk, signal out of focus, no correlation to vesicles), weakly the plasma membrane (arrow), and also variously sized droplets of PFC (white asterisks). Note that brief exposure with FM 1-43 does not stain intracellular structures, as compared to Figure 2. A differentiation between surfactant and PFC is here difficult, but can be made according to the differential appearance in DIC (PFC = indented hollow structures, white asterisk; compare with arrowhead), or by the difference in emission intensities (Inset: at largely reduced exposure times, surfactant appears much brighter than PFC, small arrow). Droplets of PFC may be distant from the cells (red asterisk), or in intimated contact with the plasma membrane (white asterisk). No noticeable difference with regard to plasma membrane, cell morphology, size or perinuclear location of intracellular LBs, and presence of exocytosed surfactant could be detected between these groups.

To exclude an effect of PFC on cellular integrity, PFC incubated cells were analyzed morphologically, using conventional and differential interference contrast microscopy. No noticeable difference with regard to plasma membrane, cell morphology, size or perinuclear location of intracellular LBs, and presence of exocytosed surfactant could be detected between these groups (Figure 8).

Differential interference contrast imaging revealed that variously sized droplets of PFC remain close to the cells (on glass cover slips). According to microscopic analysis it seem as if the droplets were not inside the cells, however, an uptake of PFC droplets cannot be excluded (white and red asterisks in Figure 8). Droplets were morphologically distinct from LBs as they appeared homogeneous, larger and endowed by regular borders when viewed by bright field illumination (not shown).

### Membrane permeability

The reduction in spontaneous and Ionomycin-induced surfactant exocytosis by PFC could be explained by a PFC-induced decrease in membrane permeability. To study the effect of PFC on membrane permeability, the uptake of the styryl dye FM 1-43 into ATII-cells was measured (Inset Figure 2). Incubation with PFD and PP2 was associated with a faster increase in FM 1-43 intensity than in not-PFC incubated cells.

In principle, this could be explained by remaining PFC droplets near the cells, as the images in Figure 8 would suggest. However, and in contrast to the glass surfaces used for microscopy, washout of PFC from plastic wells was always complete. Furthermore, even in the case PFC would remain within the wells despite washout, their staining behaviour is entirely different than those of cells: droplets of PFC stained with FM 1-43 very rapidly, with a time constant (τ) of 10 min (not shown), in contrast to several hours as seen in Figure 8.

Thus, it seems as if PFC increase the membrane permeability of the cells. However, the higher endpoints of FM 1-43 fluorescence in Figure 2 could also be explained by an increase in endocytosis, leading to an additional uptake of dye via this route. To exclude this, experiments were performed using LY as fluid phase marker of endocytosis. A time-dependent increase in LY fluorescence within each tested group was found, lasting for several hours after application (data not shown). Neither the increase in intracellular LY concentration nor the final concentration of intracellular LY was affected by PFC incubation (Table 2), suggesting that PFC do not affect endocytosis.

### Membrane fluidity and cell viability

Surfactant exocytosis requires a fusion of lamellar bodies with the cellular membrane. The process is potentially affected by a low fluidity of the cellular membrane. Therefore, the effect of PFC on membrane fluidity was measured. Fluorescence anisotropy (*r*_*ss*_) in PFC incubated cells was similar to untreated control cells (PFD = 0.105 ± 0.03; PP2 = 0.102 ± 0.03; untreated control cells = 0.119 ± 0.03). The data suggest that neither PFC with the low nor with high vapor pressure affect membrane fluidity.

Finally, the effect of PFC on surfactant exocytosis could be due to an effect on cellular viability. According to the Trypan Blue exclusion tests none of the PFC did affect the viability (Table 2).

## Discussion

The present study was performed to test whether PFC with different vapor pressures affect spontaneous and induced surfactant exocytosis in primary cultures of ATII cells. According to the present data, PFC inhibited spontaneous exocytosis, an effect that is independent from vapor pressure. Secondly, PFC with low vapor pressure reduced Ionomycin-induced exocytosis, but did not alter PMA- or ATP-induced exocytosis. PFC with high vapor pressure increased ATP-induced, but decreased Ionomycin-induced surfactant exocytosis. Finally, both PFC slightly increased the permeability of cellular membrane of ATII cells, but did not alter the membrane fluidity, endocytosis or cell viability.

Incubation with PFC was associated with a higher intracellular LTG concentration. Because LTG incubation was performed in the absence of PFC (= after washout), any direct effect of PFC on dye loading or dye fluorescence can be excluded. A most likely explanation for the higher fluorescence in the PFC-exposed groups is a larger number of vesicles that were present after this 6 hrs period due to a PFC-induced reduced rate in spontaneous exocytosis. This assumption is supported by results of the dynamic measurements of spontaneous surfactant exocytosis. These findings would partially be in contrast to previously published data by our group showing an increase in surfactant exocytosis after PFC incubation [11]. However, the increased surfactant exocytosis was found after short time exposure whereas an increase in surfactant synthesis was found after long time exposure. Thus, the higher amount of LTG could be explained by an increase in vesicle number together with a decrease in spontaneous exocytosis. Alternatively, an increase in vesicle volume due to PFC as reported by Van Eden [25] has not been found in our cells (Figure 8).

In the present study, most results were found after 6 hrs of PFC exposure. At shorter exposure times (2 and 4 hrs, respectively), no effects were found (data not shown). Thus, it seems as if a certain contact time of PFC with cells is required to show effects. For ATP and PMA treated cells, the stimulatory effect of PFC may even not be accomplished to a full extent after this time. These data are in accordance with work from other groups [15,26]. Obraztsov et al. found a time dependent uptake of PFC into cellular membranes with maximum uptake after more than 6 hours of incubation [15]. Furthermore, in most of the *in vivo *investigations a PFC effect was found after several hours of PFC incubation. Nevertheless, some other data suggest a PFC effect within a few minutes [27] or hours [11]. Fernandez and co-workers found non-specific effects on cellular activation within 5 minutes of incubation [27]. Thus, it seems as if two different PFC effects can be distinguished: a rapid effect that acts within minutes, and a more prolonged effect that requires several hours of incubation. Whereas PFC stimulated the ATP and PMA response, they decreased the Ionomycin effect after 6 hrs PFC-incubation. The explanation thereof is difficult, in particular because exocytosis is a complex process, comprising a number of different physical (e.g. vesicle translocation, disassembly of the cortical actin clamp, diffusion processes), chemical (e.g. receptor/agonist interactions, lipid merger and compartment mixing, SNARE interactions) and enzymatic (e.g. activation of PLC, PKC, CaCM-PK and subsequent phosphorylation reactions) steps, and up to now, the limiting factors are still not well defined [22,28-30]. A further complication arises with the use of PFC: Due to their inert chemical nature, they cannot be labeled, and mapping of their intracellular distribution, compartmentalization, or interaction with specific intracellular targets is almost impossible.

However, the prime target of PFC is undoubtedly the plasma membrane, which is the structure of the cell where the final and decisive steps of exocytosis occur. Chemical alterations or changes in the physical state of the plasma membrane may, therefore, entail profound effects on exocytosis. To give only one example, depletion of cholesterol in the plasma membrane of ATII cells, which disrupts SNARE association with lipid rafts, inhibited exocytosis significantly [31]. Obraztsov et al. described an uptake of PFC into cellular membranes that was associated with a stabilization of the cellular membrane [15]. In an attempt to elucidate such possible PFC-effects on the plasma membrane, a multiwell assay system was developed to test for changes in membrane permeability. The test is based on the translocation of the fluorophore FM 1-43 from the outer leaflet of the plasma membrane, into which it rapidly inserts, to internal sites. If endocytosis as a transport mechanism can be ruled out, which we did by using Lucifer Yellow, the rate of internalization depends on the fluidity/viscosity of the cell membrane. Changes in fluidity/viscosity, at constant temperature, change the thermal fluctuations and molecular motions of membrane lipids according to standard thermodynamic theories [32]. This, in turn, can be measured indirectly by the permeation properties of amphiphatic molecules like FM 1-43 and others [33] for which strong diffusional restrictions exist. Our results demonstrated an increased rate of FM 1-43 entry into PFC-treated cells. Since it was excluded that PFC change the rate of endocytosis, or the plasma membrane integrity (by the Trypan Blue exclusion test), we conclude that the increased rate of FM 1-43 reflects membrane perturbations due to the incorporation of PFC molecules. The increase of permeability does not explain the reduced spontaneous surfactant exocytosis. Several reports exist, though with some conflicting conclusions, that plasma membrane fluidity modulates exocytosis in various cells. Benzyl alcohol, for example, increases membrane fluidity and increases exocytosis in some, but not all cells, whereas a concomitant inhibition on endocytosis was noted [34]. Since PFC were also internalized by ATII cells [11] stability of cellular membranes and intracellular organelles could be affected. Obraztsov et al. [15] suggest an increased membrane stability due to PFC. The increase in membrane stability could lead to reduced fusion of LBs with plasma membrane and could thus explain reduced surfactant exocytosis.

Increase of FM 1-43 entry into cells was not reflected by a change in membrane fluidity of PFC treated cells as measured by fluorescence anisotropy. This discrepancy may indicate an increase in permeability independent of fluidity: by a change in phospholipid packing, by a change in lipid composition, or by a change in the lipid compression state. It also has to be considered that anisotropy measurements on cell suspensions are *per se *quite insensitive to small changes in fluidity. Although the mean values of PFC treated cells were lower than controls (indicating higher fluidity; Table 2), even 6 repetitions did not lead to a significant difference. However, this might indicate a higher sensitivity of the FM 1-43 method than anisotropy measurements and/or more appropriate experimental conditions used in the former method (adherent ATII cells and 37°C).

Surfactant exocytosis *in vivo *occurs constitutively, and is additionally activated by a number of different physical and chemical factors. Of the stimuli which are now considered as the two most physiological ones (stretch of the alveolar epithelium and paracrine action of ATP on P2Y_2 _receptors), only ATP can be tested without considerable technical interventions. Others, like the tumor promoter PMA, are non-physiological, but frequently used as this substance freely passes cellular membranes and directly activates PKC. Ionomycin is a research tool to model calcium-triggered exocytosis because the prime effect of this ionophor is a specific and pronounced increase in the cytosolic calcium concentration.

The inhibition of Ionomycin-induced exocytosis is difficult to explain. The two most obvious explanations, a partial depletion of intracellular Ca^2+ ^stores, and/or a diminished diffusion of Ionomycin to these internal stores due to PFC, are not likely. If the Ca^2+ ^stores would be depleted, then also the ATP response should be diminished, which was not the case, and if diffusion of Ionomycin is reduced, than this should also apply for PMA, which was obviously also not the case. Thus, we do not have a conclusive explanation why the PFC effect is different in ATP/PMA and Ionomycin treated cells.

In the present study, ATP- and PMA-induced surfactant exocytosis was significantly increased by incubation with PFC with highest vapor pressure. In contrast, the inhibitory effect on Ionomycin-induced exocytosis was most prominent in PFD, the PFC with low vapor pressure. Obraztsov et al. showed that the membrane stabilizing effect of PFC is most prominent in PFC with the lowest lipid solubility [15]. Thus, it could be speculated, that the reduced exocytosis is due to the PFC incorporation of PFC with low vapor pressure into the cellular membrane and inhibited depletion of intracellular Ca^2+ ^stores.

Cellular experiments with PFC are always very difficult due to the unique properties of PFC. A potential source of variability could be the PFC-suspension itself: Due to the poor miscibility and rapid phase separation between medium/PFC during preparation of the suspensions, the effective concentration of PFC may differ considerably from the initially applied 10%. By analyzing the PFC suspensions, we found that the size distribution of PFC-droplets is considerable, but not different between the PFC used. Furthermore, the total amount of droplets did not differ between the PFC and were stable over time, demonstrating consistent and comparable measurement conditions.

In summary, the present study supports the assumption that PFC interact with cellular membranes, an effect that requires several hours of incubation and leads to a slight increase in membrane permeability that in turn alters the rate of spontaneous and stimulated exocytosis.

## Abbreviations

(ATP): Adenosintriphosphat; (ATII cells): Alveolar Type II cells; (BB): Brilliant Black; (CaCM-PK): Ca^2+^/calmodulin-dependent protein kinases; (DIC): Differential interference contrast microscopy; (DMEM): Dulbecco's Modified Eagle's medium; (DPH): 1,6-diphenyl-1,3,5-hexatriene; (FCS): Fetal calf serum; (HEPES): (4-(2-hydroxyethyl)-1-piperazineethanesulfonic acid; (LBs): Lamellar Bodies; (LTG): LysoTracker Green; (LY): Lucifer Yellow; (PFC): Perfluorocarbons; (PFD): Perfluorodecalin; (PKC): Protein kinase C; (PMA): Phorbol 12-myristate 13-acetate; (PP2): Flutec™ PP2 (Perfluoromethylcyclohexan); (RFU): Relative fluorescence units; (SNARE): Soluble NSF Attachment Receptors.

## Competing interests

The authors declare that they have no competing interests.

## Authors' contributions

AW contributed to the conception and design of the study, calculated the data and wrote in part the manuscript. IH, NH and AR performed all experiments. TH contributed substantially to experimental design, data interpretation and assisted in drafting the manuscript. MR was responsible for the objective and general interpretation of this study. All authors read and approved the final manuscript.

## References

[B1] Leach CL Greenspan JS Rubenstein SD Shaffer TH Wolfson MR Jackson JC deLemos RA Fuhrman BP Partial liquid ventilation with perflubron in premature infants with severe respiratory distress syndrome N Engl J Med 1996 335 761 767 10.1056/NEJM199609123351101 8778584

[B2] Rotta AT Steinhorn DM Partial liquid ventilation reduces pulmonary neutrophil accumulation in an experimental model of systemic endotoximia and acute lung injury Crit Care Med 1998 26 1707 1715 10.1097/00003246-199810000-00026 9781729

[B3] Rotta AT Gunnarsson B Hernan LJ Fuhrman BP Steinhorn DM Partial liquid ventilation influences pulmonary histopathology in an animal model of acute lung injury J Crit Care 1999 14 84 92 10.1016/S0883-9441(99)90019-9 10382789

[B4] Burkhardt W Koehne P Wissel H Graf S Proquitté H Wauer RR Rüdiger M Intratracheal perfluorocarbons diminish LPS induced increase in systemic TNFa Am J Physiol Lung Cell Mol Physiol 2008 294 L1043 L1048 10.1152/ajplung.00125.2007 18359887

[B5] Modell JH Gollan F Giammona ST Effect of fluorocarbon liquid on surface tension properties of pulmonary surfactant Chest 1970 57 263 265 10.1378/chest.57.3.263 5417668

[B6] Ruefer R Surfactant and alveolar surface forces after breathing of an inert fluorinated liquid Fed Proc 1970 29 1813 1815 5457592

[B7] Ruefer R Spitzer HL Liquid ventilation in the respiratory distress syndrome Chest 1974 66 29S 30S 10.1378/chest.66.1_Supplement.29S 4495151

[B8] Gladstone IM Ray AO Salafia CM Pérez-Fontán J Mercurio MR Jacobs HC Effect of artificial surfactant on pulmonary function in preterm and full-term lambs J Appl Physiol 1990 69 465 472 2121700 10.1152/jappl.1990.69.2.465

[B9] Mercurio MR Fiascone JM Lima DM Jacobs HC Surface tension and pulmonary compliance in premature rabbits J Appl Physiol 1989 66 2039 2044 2745272 10.1152/jappl.1989.66.5.2039

[B10] Steinhorn DM Leach CL Fuhrman BP Holm B Partial liquid ventilation enhances surfactant phospholipid production Crit Care Med 1996 24 1252 1256 10.1097/00003246-199607000-00031 8674344

[B11] Rüdiger M Wissel H Ochs M Burkhardt W Proquitté H Wauer RR Stevens P Rüstow B Perfluorocarbons are taken up by isolated type II pneumocytes and influence its lipid synthesis and secretion Crit Care Med 2003 31 1190 1196 10.1097/01.CCM.0000060008.96029.7D 12682492

[B12] Rüdiger M Wendt S Köthe L Burkhardt W Wauer RR Ochs M Alterations of alveolar type II cells and intraalveolar surfactant after bronchoalveolar lavage and perfluorocarbon ventilation. An electron microscopical and stereological study in the rat lung Resp Res 2007 8 40 10.1186/1465-9921-8-40 PMC1892019 17550584

[B13] Haller T Ortmayr J Friedrich F Völkl H Dietl P Dynamics of surfactant release in alveolar type II cells Proc Natl Acad Sci USA 1998 95 1579 1584 10.1073/pnas.95.4.1579 9465058 PMC19102

[B14] Rooney SA Regulation of surfactant secretion Comp Biochem Physiol A Mol Integr Physiol 2001 129 233 243 10.1016/S1095-6433(01)00320-8 11369548

[B15] Obraztsov VV Neslund G Kornbrust E Flaim S Woods CM In vitro cellular effects of perfluorochemicals correlate with their lipid solubility Am J Physiol 2000 278 L1018 L1024 10.1152/ajplung.2000.278.5.L1018 10781433

[B16] Dietl P Haller T Mair N Frick M Mechanisms of surfactant exocytosis in alveolar type II cells in vitro and in vivo News Physiol Sci 2001 16 239 243 11572929 10.1152/physiologyonline.2001.16.5.239

[B17] Wemhöner A Frick M Dietl P Jennings P Haller T A fluoresecent microplate assay for exocytosis in alveolar type II cells J Biomol Screening 2006 11 286 295 10.1177/1087057105285284 16699129

[B18] Dobbs LR Gonzalez R Williams MC An improved method for isolating type II cells in high yield and purity Am Rev Respir Dis 1986 134 141 145 3637065 10.1164/arrd.1986.134.1.141

[B19] Rüdiger M Köpke U Prösch S Rauprich P Wauer RR Herting E Effects of perfluorocarbons and perfluorocarbons/surfactant-emulsions on growth and viability of group B streptococci and Escherichia coli Crit Care Med 2001 29 1786 1791 10.1097/00003246-200109000-00022 11546986

[B20] Betz WJ Mao F Smith CB Imaging exocytosis and endocytosis Curr Opin Neurobiol 1996 6 365 371 10.1016/S0959-4388(96)80121-8 8794083

[B21] Haller T Dietl P Pfaller K Frick M Mair N Paulmichl M Hess MW Fürst J Maly K Fusion pore expansion is a slow, discontiuous, and Ca^2+^-dependent process regulating secretion from alveolar type II cells J Cell Biol 2001 155 279 289 10.1083/jcb.200102106 11604423 PMC2198834

[B22] Chander A Chen XL Naidu DG A role for diacylglycerol in annexin A7-mediated fusion of lung lamellar bodies Biochim Biophys Acta 2007 1771 1308 1318 17765009 10.1016/j.bbalip.2007.07.004 PMC2100037

[B23] Shinitzky M Barenholz Y Fluidity parameters of lipid regions determined by fluorescence polarization Biochim Biophys Acta 1978 515 367 394 365237 10.1016/0304-4157(78)90010-2

[B24] Sousa C Nunes C Lucio M Ferreira H Lima JL Tavares J Cordeiro-da-Silva A Reis S Effect of nonsteroidal anti-inflammatory drugs on the cellular membrane fluidity J Pharm Sci 2008 97 3195 3206 10.1002/jps.21218 17990311

[B25] van Eeden SF Klut ME Leal MA Alexander J Zonis Z Skippen P Partial liquid ventilation with perfluorocarbon in acute lung injury. Light and transmission electron microscopy studies Am J Respir Cell Mol Biol 2000 22 441 450 10745025 10.1165/ajrcmb.22.4.3717

[B26] Woods CM Neslund G Kornbrust E Flaim SF Perflubron attenuates neutrophil adhesion to activated endothelial cells in vitro Am J Physiol 2000 278 1008 1017 10.1152/ajplung.2000.278.5.L1008 10781432

[B27] Fernandez R Sarma V Younkin E Hirschl RB Ward PA Younger JG Exposure to perflubron is associated with decreased Syk phosphorylation in human neutrophils J Appl Physiol 2001 91 1941 1947 11641328 10.1152/jappl.2001.91.5.1941

[B28] Dietl P Haller T Exocytosis of lung surfactant: from the secretory vesicle to the air-liquid interface Annu Rev Physiol 2005 67 595 621 10.1146/annurev.physiol.67.040403.102553 15709972

[B29] Chander A Sen N Spitzer AR Synexin and GTP increase surfactant secretion in permeabilized alveolar type II cells Am J Physiol Lung Cell Mol Physiol 2001 280 L991 L998 11290524 10.1152/ajplung.2001.280.5.L991

[B30] Wang P Chintagari NR Narayanaperumal J Ayalew S Hartson S Liu L Proteomic analysis of lamellar bodies isolated from rat lungs BMC Cell Biol 2008 9 34 10.1186/1471-2121-9-34 18577212 PMC2459160

[B31] Chintagari NR Jin N Wang P Narasaraju TA Chen J Liu L Effect of cholesterol depletion on exocytosis of alveolar type II cells Am J Respir Cell Mol Biol 2006 34 677 687 10.1165/rcmb.2005-0418OC 16439800 PMC2644229

[B32] Coster HGL The Physics of Cell Membranes Journal of Biological Physics 2003 29 363 399 10.1023/A:1027362704125 PMC3456180 23345856

[B33] Kubina M Lanza F Cazenave JP Laustriat G Kuhry JG Parallel investigation of exocytosis kinetics and membrane fluidity changes in human platelets with the fluorescent probe, trimethylammonio-diphenylhexatriene Biochim Biophys Acta 1987 901 138 146 10.1016/0005-2736(87)90265-3 3593721

[B34] Giocondi MC Mamdouh Z Le Grimellec C Benzyl alcohol differently affects fluid phase endocytosis and exocytosis in renal epithelial cells Biochim Biophys Acta 1995 1234 197 202 10.1016/0005-2736(94)00284-V 7696294

